# Spatial association between outdoor air pollution and lung cancer incidence in China

**DOI:** 10.1186/s12889-019-7740-y

**Published:** 2019-10-26

**Authors:** D. F. Xing, C. D. Xu, X. Y. Liao, T. Y. Xing, S. P. Cheng, M. G. Hu, J. X. Wang

**Affiliations:** 1 State Key Laboratory of Resources and Environmental Information System, Institute of Geographic Sciences and Natural Resources Research, Chinese Academy of Sciences, Beijing, China; 2 School of Information Engineering, China University of Geosciences, Beijing, 100083 China; 3 Key Laboratory of Land Surface Pattern and Simulation, Beijing Key Laboratory of Environmental Damage Assessment and Remediation, Institute of Geographic Sciences and Natural Resources Research, Chinese Academy of Science (CAS), Beijing, 100101 China; 4 Key Laboratory of Water and Sediment Sciences, Ministry of Education; Department of Environmental Engineering, Peking University, Beijing, 100871 China

**Keywords:** Lung cancer, Outdoor air pollution, GeoDetector, Spatial association, Smoking

## Abstract

**Background:**

Lung cancer is the most common cancer in China. Previous studies have indicated that lung cancer incidence exhibits remarkable spatial heterogeneity, and lung cancer is related to outdoor air pollution. However, the non-linear spatial association between outdoor air pollution and lung cancer incidence in China remains unclear.

**Methods:**

In this study, the relationships between the lung cancer incidence of males and females from 207 counties in China in 2013 with annual concentrations of PM2.5, PM10, SO_2_, NO_2_, CO and O_3_ were analysed. GeoDetector q statistic was used for examining the non-linear spatial association between outdoor air pollution and incidence of lung cancer.

**Results:**

An apparent spatial and population gender heterogeneity was found in the spatial association between outdoor air pollution and lung cancer incidence. Among the six selected pollutants, SO_2_ has the greatest influence on lung cancer (*q* = 0.154 in females) in north China. In the south, each selected pollutant has a significant impact on males or females, and the mean q value in the south is 0.181, which is bigger than that in the north (*q* = 0.154). In addition, the pollutants have evident non-linear interaction effects on lung cancer. In north China, the interaction between SO_2_ and PM2.5 is the dominant interaction, with q values of 0.207 in males and 0.334 in females. In the south, the dominant interactive factors are between SO_2_ and O_3_ in males and between SO_2_ and CO in females, with q values of 0.45, 0.232 respectively. Smoking is a substantial contributor to lung cancer among men, either in South or North China, with q value of 0.143 and 0.129 respectively, and the interaction between smoking and air pollutants increases this risk.

**Conclusions:**

This study implies that the influence of SO_2_ and PM2.5 on lung cancer should be focused on in north China, and in the south, the impact of O_3_ and CO as well as their interaction with SO_2_ need to be paid more attention. Smoking, particularly in men, remains a significant risk factor for lung cancer in both North and South China.

## Background

Lung cancer is one of the most serious malignancies which threaten human life; as the leading cause of cancer deaths worldwide, it accounts for about 26% of all cancers and is ranked as the deadliest cancer among males and the second deadliest cancer among females [1, 2]. Despite the overall 5-year survival rate for lung cancer has slightly improvement over time, it still remains poor, only with 16.8% in 2004 [3].

The incidence of lung cancer in China is at a high level relative to that in the rest of the world, and it is also the highest incidence of malignant tumours in China [4]. According to the Third National Causes of Death Sampling Survey (2004–2005) of China, the mortality of lung cancer increased significantly from 5.47/10^5^ in the 1970s to 30.83/10^5^ in 2004–2005 [5], and in the next few years, lung cancer mortality remained high [6]. The severity of China’s lung cancer problem results in a considerable economic burden every year. From 1996 to 2011, the direct medical expenses for lung cancer cases amounted to $1517–$4018, with an average annual growth rate of 2.2% [7].

Previous studies have shown that smoking status, family history and other factors (e.g. indoor air pollution, radon exposure) have significant effects on lung cancer [3, 8]. As one of the leading risk factors for cancer, smoking contributes a large proportion of the lung cancer burden, especially among men. One comparative risk assessment conducted in mainland China indicated that the population-attributable fractions of lung cancer for smoking in men is approximately 55.1%, about five times that of women [9].

Recently, an increasing number of studies have reported positive associations between risk of lung cancer and outdoor air pollution [10, 11]. Since the 1990s, outdoor air pollution has been a top environmental problem in China, accompanied by the rapid development of industry and urbanisation [12]. In 2000, the concentrations of air pollutants in more than half the country’s cities were over Chinese Grade II standards (hazardous to health; highest level is Grade VI). Severe air pollution problems pose a great threat to health [13]. In China, the mortality attributed to air pollution is higher than that linked to alcohol and drug use and ranks as the fourth cause of death [14].

Evidence of the association between exposure to outdoor air pollution and lung cancer continues to accumulate [15–17], but the understanding of which pollutants control or influence the incidence (or mortality) and its spatial variation remains an unsolved problem. Inhalable airborne particles (PM2.5, PM10) have a statistical association with lung cancer [18], and each 10 μg/m^3^ increase in PM2.5 concentration is correlated with a 15–27% increase in lung cancer mortality [19]. Meanwhile, gaseous pollutants, such as sulphur dioxide (SO_2_), ozone (O_3_), carbon monoxide (CO), and nitrogen dioxide (NO_2_) have also been examined for potential relationships with lung cancer [20–22]. However, results from previous studies are sometimes inconsistent, an important cause may be the fundamental regional spatial association between various pollutants and lung cancer.

China covers a vast territory, and its northern and southern regions present an obvious spatial heterogeneity in climatic conditions, economic and industry structure. Their air pollutants also have apparent regional characteristics. For example, the concentrations of PM2.5, PM10, SO_2_ and CO in north China are significantly higher than those in south China [23, 24].

In this study, GeoDetector [25] was used to quantitatively assessing the determinant power of principle air pollutants and smoking on lung cancer, analysing the interaction of these factors in North and South China. Their various influences and associations with lung cancer in these regions were then analysed and discussed.

## Methods

### Study area

China, which has high lung cancer burden in the world, is the largest developing country located in the eastern part of Asia and the west coast of the Pacific Ocean (3°52′–53°33′ N, 73°30′–135°3′ E). It was divided into the north and the south regions using a prominent geographical boundary created by the Qinling Mountains and the Huai River line. Apparent differences of climate characteristics, economic and industry structure exists in the north and the south regions. For example, north China is dominated by temperate continental climate, it has long and dry winter, while the south China was dominated by subtropical monsoon climate, it has higher temperature and more abundant precipitation. The heavy industries hold the important position in the north for it has the abundant energy resources (eg, coal, petroleum), while the south pays more attention to the development of light industry.

### Data of lung cancer

**Fig. 1:**
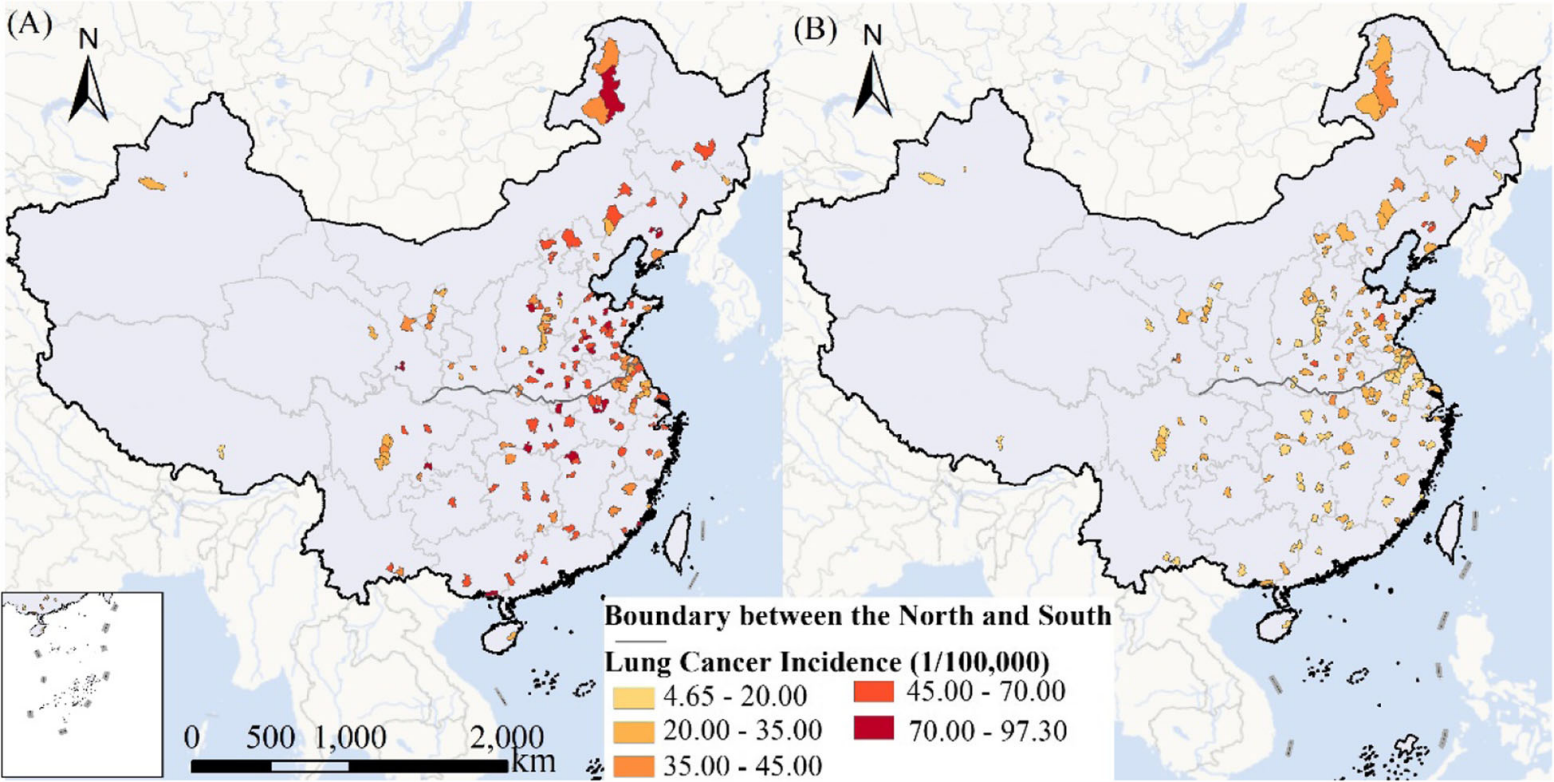
Lung cancer incidence in (A) males and (B) females in 207 counties of China in 2013

The female and male lung cancer incidence data in 2013 in 207 counties of China were obtained from the *Annual Report of Cancer Registration in China 2016* [26] (Fig. 1). The data has been evaluated for integrity and reliability in accordance with the Guidelines for Cancer Registration in China, and has been released as official data by the National Cancer Centre. The dataset covered 255 cities and counties from all 31 provinces in mainland China, with a total survey population of 227 million, accounting for about 20% of the country’s total population. After geocoding each county, 207 were matched in the county map. The incidence of lung cancer was adjusted by age structure.

There are apparent climatic, industry structure and socio-economic differences on either side of the Qinling Mountains and the Huai River, which serves as the boundary between north and south China [27]. These 207 counties distributed in 31 provinces of China, among them, 102 are located in north China and 105 are located in south China. In north China, the total incidence of lung cancer is 38.91/10^5^, the lung cancer incidence among males and females are 51.69/10^5^ and 26.13/10^5^ respectively. In south China, the total incidence of lung cancer is 35.51/10^5^, the lung cancer incidence among males and females are 51.53/10^5^ and 19.48/10^5^ respectively (Table 1). In this study, the spatial pattern of lung cancer incidences in north and south China as well as in different genders were analysed.

**Table 1:** Statistical information for lung cancer incidence in different genders in north and south China

Lung cancer Incidence (1/100,000)	Max	Min	Mean	Standard deviation
Males in north China	118.27	20.50	51.69	17.10
Females in north China	66.93	11.63	26.13	9.96
Males in south China	97.3	4.65	51.53	16.53
Females in south China	36.6	0.00	19.48	6.29

### Air pollution data

Previous studies have indicated that the air pollution index, e.g. PM2.5, PM10, SO_2_, O_3_, CO, and NO_2_ has an important effect on lung cancer in China [28]. Epidemiological studies have shown that air pollutants, such as PM2.5, PM10 and O_3_, contribute to oxidative stress in the pulmonary system, thus potentially increasing the production of mediators of pulmonary inflammation and initiating or promoting mechanisms of carcinogenesis [29]. In the current study, data about these factors were collected for an analysis of their contribution to the spatial variation of lung cancer.

The China National Environmental Monitoring Centre published air pollution data for mainland China in May 2014, recording a concentration of six air pollutants—PM2.5, PM10, SO_2_, O_3_, CO, and NO_2_—every hour throughout 936 national monitoring stations, which were examined by quality assurance procedures. In this study, the annual average concentration of each of these six lung cancer pollutants from May 2014 to May 2015 was calculated and analysed (Table 2). Regression-kriging was used to interpolate the air pollution of these 207 counties based on air pollution data from the 936 monitoring stations.

**Table 2:** Statistical information for each annual pollutant

Factor	Abbreviation	Max	Min	Mean	Standard deviation
Fine particulate (μg/m^3^)	PM2.5	139.22	14.40	64.18	21.05
Inhalable particles (μg/m^3^)	PM10	235.32	37.46	110.87	37.67
Sulfur dioxide (μg/m^3^)	SO_2_	192.71	1.92	35.54	25.05
Ozone (μg/m^3^)	O_3_	98.44	0.00	50.66	15.08
Carbon monoxide (mg/m^3^)	CO	3.27	0.41	1.26	0.46
Nitrogen dioxide (μg/m^3^)	NO_2_	89.42	7.42	39.12	13.87

Note: μg/m^3^: 1 microgram per cubic meters; mg/m^3^: 1 mg per cubic meters

#### Fine particulate (PM2.5)

The impact of fine particulate matter (PM2.5) on lung cancer has been widely discussed in many previous studies [30]. A number of researchers have found that PM2.5 is positively correlated with the incidence of lung cancer [31]. Following previous work, the present research used PM2.5 to assess the risk of lung cancer incidence.

In general, the average concentration of PM2.5 in the north (70.51 μg/m^3^) is higher than that in the south (57.86 μg/m^3^). Among the 10 counties with the highest concentration of PM2.5 in the north, 6 of them are located in Hebei province, which is the typical province dominated by industry adjacent to Beijing in south China. The most polluted PM2.5 areas are the north China plain, following with the Sichuan basin.

#### Inhalable particles (PM10)

Compared with the association between PM2.5 and lung cancer, that between PM10 and lung cancer has been less studied. Most previous studies have stated that the correlation of PM10 with lung cancer is not as strong as that of PM2.5. Although a positive association between PM10 and lung cancer incidence has been found by some studies [30], it still requires further research.

The average concentration of PM10 in the north and south is 131.72 μg/m^3^ and 90.02 μg/m^3^, respectively. The spatial distribution of high concentration areas of PM10 is approximately the same as that of PM2.5. Counties in north China plain also took the important position with the average concentration of approximately 180 μg/m^3^. In the south China, the regions with high concentration of PM10 are in the southwest and eastern China.

#### Sulfur dioxide (SO_2_)

SO2, similar to PM, is a major pollutant in China. Given that they are both derived primarily from coal combustion, the effects of SO2 and PM are often considered together. Studies have shown that exposure to SO_2_ increases the risk of lung cancer, and it is estimated that a 1% increase in SO_2_ emissions can lead to 0.004 more deaths per 100,000 population due to lung cancer in China [32]. Therefore, analysing the impact of SO_2_ on different regions of China is of significance.

As a product of coal combustion, the average concentration of SO_2_ in studied counties in the north (50.56 μg/m^3^) is significantly higher than that in the south (20.50 μg/m^3^). This is consistent with the energy structures in these two regions, in north China, the consumption of coal is higher than in south due to the long winter and industrial consumption. High concentration areas of SO_2_ are mainly located in the north China plain and northeast China, where the heavy industry based on coal-consuming is the dominant industry.

#### Ozone (O_3_)

The effect of O_3_ on lung cancer is varied in different studies. Most existing studies are concerned about the impact of O_3_ on lung cancer mortality rate. Some studies have found a strong negative correlation between O_3_ and lung cancer [33], whereas some found no evidence of such association [20, 34]. The different results may be caused by variations in the study areas or methods.

Among the six studied pollutants, O_3_ is the only one whose concentration in the south is higher than that in the north, with 49.97 μg/m^3^ in north and 50.35 μg/m^3^ in south. The geographical distribution of O_3_ has strong spatial heterogeneity, it is mainly concentrated in Sichuan basin in southwest and eastern China, although the average concentration of O_3_ in the north is lower than that in the south, there are still high concentrations in regions near Beijing.

#### Carbon monoxide (CO)

Few studies have explored the association between CO and lung cancer in detail; most related studies analysed it with NO_2_, SO_2_ and other pollutants. Some works have found that similar to NO_2_ and SO_2_, CO is significantly associated with lung cancer incidence [35], whereas others found no association [36]. Thus, further studies are needed.

The average concentration of CO in total 102 studied counties in the north is 1.46 mg/m^3^, which is higher than that in the south (1.05 mg/m^3^). High concentrations of CO are in several provinces in the north China plain, while in the south, few areas have high concentrations of CO.

#### Nitrogen dioxide (NO_2_)

There are many studies focusing on the association between NO_2_ and lung cancer, many of which have suggested that NO_2_ has an important impact on lung cancer [36, 37]. However, there remains inconsistencies about the role of NO_2_ in lung cancer incidence in men and women [35].

The average concentration of NO_2_ in total 102 studied counties in the north is 41.52 μg/m^3^, which is higher than that in the south (36.72 μg/m^3^). High concentrations of NO_2_ are mainly in regions near Beijing.

### Smoking rate data

The smoking rate dataset came from a serial cross-sectional National Health Services Survey (NHSS) organized by the National Health Commission (NHC) of the People’s Republic of China. Smoking status was surveyed of the entire population of 31 provinces, autonomous regions, and municipalities in mainland of China. In this smoking rate dataset, the proportion of current smokers in 2008 was 24.9%. For men, prevalence was 48.4%, while it was less than one-tenth of that (2.3%) for women [38]. Therefore, it mainly represent the smoking status of males. In the current study, smoking rate data in study region was obtained using geostatistical-based downscaling method based on the dataset [39–41], and then was used to analyse its association with lung cancer in men and the effect of interaction with air pollutants.

### GeoDetector

In this study, GeoDetector was used for assessing the influence of select air pollution factors on lung cancer incidence in north and south China. GeoDetector is a spatial variance analysis method that explains non-linear associations between potential factor and spatial phenomenon [42, 43]. The method was first proposed for assessing the spatial association between neural tube defect occurrences and their risk factors [25], and it has since been widely applied to such areas as health, environment and geography [44, 45]. In GeoDetector, factor detector and interaction detector are two commonly used functions.

In this study, the factor detector was used for detecting the spatial association between incidence of lung cancer and potential air pollution factors. To introduce the method briefly, we assume that X is a risk factor (e.g., PM2.5, SO_2_, CO, etc), which was used to test the spatial association with lung cancer incidence. The core underlying assumption of the Geodetector q statistic is that, if X (explanatory variable) causes Y (explained variable), their spatial distribution will be consistent. In terms of this study, if the air pollutant (X) has an important influence on lung cancer rate (Y), then the spatial distribution of X would be similar to that of lung cancer occurrence. The determinant power of X was quantified by the q statistic, where the range of q is the interval [0, 1]. In the calculation process, X was first divided into several different strata, then the spatial association between X and Y was analyzed by comparing variances between the strata. The spatial correspondence between X and incidence of lung cancer could be measured by the q statistic which is defined as.

1 $$ \mathrm{q}=1-\frac{\sum \limits_{h=1}^S{N}_h{\sigma}_h^2}{N{\sigma}^2}=1-\frac{SSW}{SST} $$

2 $$ {\displaystyle \begin{array}{l} SSW=\sum \limits_{h=1}^S{N}_h{\sigma}_h^2\\ {} SST=N{\sigma}^2\end{array}} $$

where *h = 1,2 … ., S* is the strata of an air pollution factor; *N*_*h*_ and *N* present the numbers of counties in stratum *h* and in the whole study counties, respectively; ${\sigma}_h^2$ and *σ*^2^ denote the variances in lung cancer incidences in stratum *h* and in the whole study area, respectively. SSW and SST are the variance of lung cancer incidence within strata *h* of factor X and the global variance of lung cancer incidence in the whole study area, respectively. The *q* value means that a factor explains *q × 100*% of the lung cancer incidence; the bigger the *q* value, the larger the non-linear association with regard to the incidence of lung cancer. The range of *q* is in the interval [0, 1]. In the study, the south and the north China were divided into three spatial strata, respectively. The stratification was implemented based on the minimum within-strata variation and maximum between-strata variation constraint [42, 43].

Non-central F-distribution was used for the test of spatial association between two factors [42], which is defined as.

3 $$ \mathrm{P}\left(1<\chi \right)=\mathrm{P}\left(\mathrm{F}<\frac{N-S}{S-1}\frac{\chi }{1-\chi}\right)=1-\alpha $$

4 $$ \upchi =\frac{1}{\sigma^2}\left[{\sum}_{h=1}^S{\overline{Y}}_h^2-\frac{1}{N}{\left({\sum}_{h=1}^S\sqrt{N_h}{Y}_h\right)}^2\right] $$

where α is the probability of q ≥ χ. ${\overline{Y}}_h$ is the mean value of stratum *h*. The null hypothesis of the test is: there presents no spatial association between two factors and the alternative hypothesis is: there exists spatial association between them, respectively. Note that if spatial distribution of lung cancer incidence is completely driven by the factor X, the within sum of variance is 0 and q = 1, while if *q* = 0, it implies that lung cancer incidence and factor X have not any spatial association with each other.

The q statistic is a method of measuring the spatial association between two variables. In this study, the q statistic was not only used to measure the spatial relationship between air pollutants or smoking rate and lung cancer in factor detector but also to evaluate the effects on lung cancer of interactions between two factors.

Interaction detector was used for identifying the interaction effect between two factors on lung cancer. Interaction detector assess the interaction effect by comparing value of q(X1 ∩ X2) with values of q(X1) and q(X2), in which X1 ∩ X2 was implemented by overlaying the two variables using GIS. The method could determine the kind of interaction (non-linear weakening, univariate weakening, bivariate enhancement, independent, non-linear enhancement) existing between two air pollution factors acting together (Table 3). The GeoDetector can be freely downloaded from http://www.geodetector.cn/.

**Table 3:** Interaction relationship between two factors

Description	Interaction
q(X1 ∩ X2) < Min(q(X1), q(X2))	Non-linear weakening
Min(q(X1), q(X2)) < q(X1 ∩ X2) < Max(q(X1), q(X2))	Univariate weakening
q(X1 ∩ X2) > Max(q(X1),q(X2))	Bivariate enhancement
q(X1 ∩ X2) = q(X1) + q(X2)	Independent
q(X1 ∩ X2) > q(X1) + q(X2)	Non-linear enhancement

Note: q(X1 ∩ X2) represents the q value from the interaction relationship between pollutants X1 and X2

## Results

### Spatial patterns

In general, the incidence of lung cancer in males was apparently higher than that in females. In 2013, the mean incidences of male and female lung cancer were 51.61/10^5^ and 22.76/10^5^, respectively. The incidences of lung cancer in north and south China only slightly differed at 38.91/10^5^ and 35.51/10^5^, respectively (Fig. 1).

### Factor detector

The results of the factor detector indicated spatial and population gender heterogeneities in the spatial association between outdoor air pollution and lung cancer incidence. In general, air pollution in south China had a greater impact on lung cancer than in the north, and males in the south were more likely to be affected by polluted air than were the females. Table 4 shows the results of the factor detector in north and south China.

**Table 4:** q values for different air pollutant factors and smoking rates in north and south China

	PM2.5	PM10	SO_2_	NO_2_	O_3_	CO	Smoking
North							
Male	0.083	0.070	0.093	0.036	0.083	0.065	0.143*
Female	0.153	0.034	0.154*	0.107	0.056	0.156	–
South							
Male	0.243**	0.210	0.140**	0.199**	0.314**	0.230**	0.129*
Female	0.110*	0.101*	0.098	0.103	0.071	0.113*	–

Note: ** 1% level of statistical significance; * 5% level of statistical significance

In north China, among the six selected pollutants, SO_2_ was the dominant factor affecting the spatial distribution of lung cancer in the region, with a q value of 0.154 (*p* value < 0.05) for the females.

In south China, PM2.5 and CO were found to be important factors that had a significant influence on lung cancer incidence in both sexes. For the males, O_3_ had the greatest explanatory power for lung cancer incidence, with a q value of 0.314. SO_2_ played a relatively weak role, with a q value of 0.140. As for the females, results showed that CO, PM2.5 and PM10 had a significant influence on lung cancer incidence, with q values of 0.113, 0.110 and 0.101 (*p* values < 0.05), respectively. Significant association between lung cancer and smoking rate was found among men, with q values of 0.143 and 0.129 in North China and South China, respectively.

### Interaction detector

**Fig. 2:**
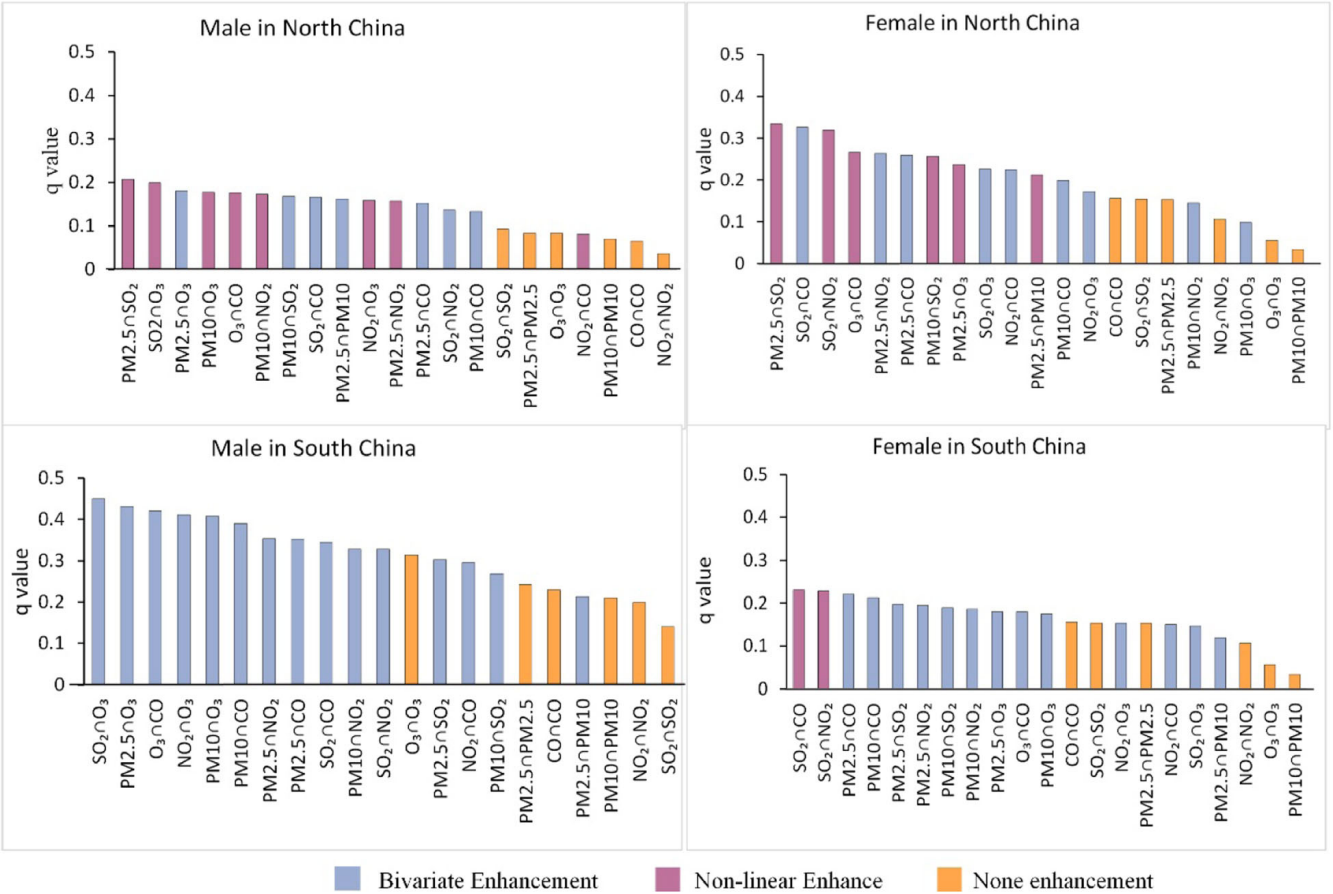
Interaction results between air pollutants

The results of the interaction detector showed that the interaction between any two pollutants enhanced the risk of lung cancer (Fig. 2).

For the males in north China, the interaction between SO_2_ and PM2.5 had the highest q value of 0.207, which is greater than the sum of the q values of the two factors, showing a relationship of non-linear enhancement, which implies there presents effect modification for the cancer. This was followed by the interaction between SO_2_ and O_3_, which had a q value of 0.199, and the interaction between PM2.5 and O_3_, which had a q value of 0.180. The dominant interaction is between SO_2_ and PM2.5.

For the females in north China, the interaction between SO_2_ and PM2.5 also had effect modification, as the highest q value (0.334) and it is greater than the sum of the q values of the two factors. This was followed by the interaction between SO_2_ and CO, which presented a q value of 0.326, and the interaction between SO_2_ and NO_2_, which showed a q value of 0.319. The interaction is between SO_2_ and PM2.5 also took the dominant role among the females in north China.

Among the males in south China, the interaction between SO_2_ and O_3_ had the highest q value of 0.45, which is greater than the maximum q value among the two factors, indicating a relationship of bivariate enhancement. This was followed by the interaction between PM2.5 and O_3_, which had a q value of 0.431, and the interaction between O_3_ and CO, which had a q value of 0.421. The dominant interaction is between SO_2_ and O_3_.

Among the females in south China, the interaction between SO_2_ and CO had the highest q value of 0.231, which is greater than the sum of the q values of the two factors, implying there presents effect modification for the cancer. This was followed by the interaction between SO_2_ and NO_2_, which had a q value of 0.229, and the interaction between PM2.5 and CO, which showed a q value of 0.221. The dominant interaction is between SO_2_ and CO.

Note that despite the dominant factor and interaction in north and south as well as in males and females are different, SO_2_ appears in all the dominant interactions, in other words, in all six selected pollutants, SO_2_ needs to be focused on no matter in south or north.

**Fig. 3:**
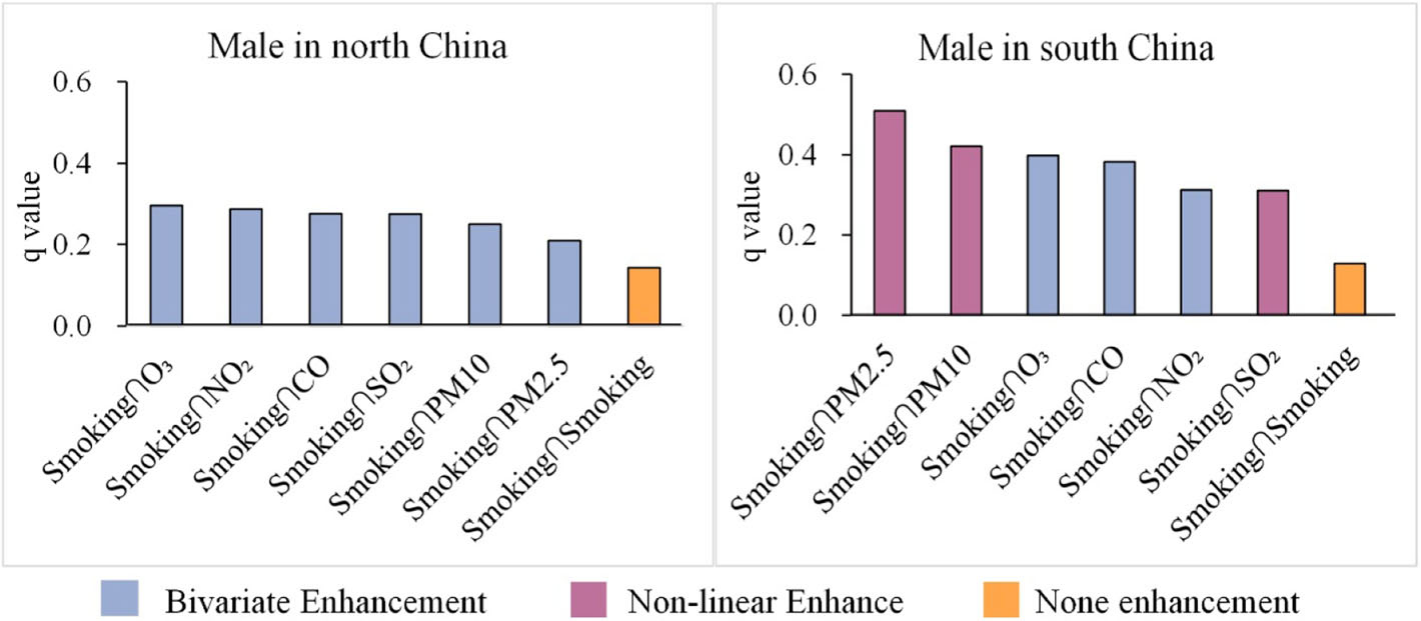
Interaction results between smoking and air pollutants

The effects of interaction between smoking and air pollutants in North and South China is shown in Fig. 3, indicating that q values of pollutants increased significantly after interacting with the smoking rate.

For males in North China, the interaction between smoking and O_3_ had the highest q value of 0.296—much greater than the q value of O_3_ alone—followed by the interaction between smoking and NO_2_, which had a q value of 0.288. The dominant interaction being between smoking and O_3_.

For males in South China, the interaction between smoking and PM2.5 had the highest q value of 0.510, which is greater than the sum of the q values of each factor individually, indicating effect modification for the cancer. This was followed by the interaction between smoking and PM10, with a q value of 0.421, and the interaction between PM2.5 and O_3_, showing a q value of 0.398. The dominant interaction in South China was between smoking and PM2.5.

## Discussion

As one of the most common malignancies in China, lung cancer poses a great threat to the health of the Chinese. Air pollution has been found to be a risk factor of lung cancer, whereas the spatial association between pollutants and lung cancer in China remains unclear. In this study, the influence of outdoor air pollution on lung cancer incidence in north and south China for different genders was analysed. These results indicated that the influence of air pollution on lung cancer presented apparent spatial and gender heterogeneities, and when smoking combined with air pollution, the risk of lung cancer greatly increased in males.

The consumption of coal in China has been higher than the global average. In 2013, the amount of coal use in China is about 2943 million tons, accounting for more than half of global total [46]. As a product of coal combustion, SO_2_ is more harmful in China than are traffic-related pollutants, such as NO_2_ [47]. One study examined the association of SO_2_ and NO_X_ with mortality in China and indicated that an increase of 10 μg/m^3^ of SO_2_ corresponded to a 4.2% (95% CI: 2.3, 6.2%) increase of lung cancer mortality, which was apparently higher than that of NO_X_ (2.7% (95% CI: − 0.9, 6.5%)) [10].

In the current research, we found a larger impact of SO_2_ on lung cancer in the north compared with that in the south. This may be because the concentration of SO_2_ in northern China is obviously higher than that in the south. One study which analysis the spatial variation in 26 cities in China reported that the average concentration of SO_2_ in northern cities is 72 μg/m^3^, almost as twice as that of southern cities [24], and more than three times the standard concentration given by the World Health Organization (WHO) air quality guidelines, suggesting that the minimum health-hazard concentration for SO_2_ is less than 20 μg/m^3^. Exposure to high levels of SO_2_ increases the risk of lung cancer in northern populations.

PM2.5 has been widely examined for its effect on lung cancer. A study in China which examined the association between lung cancer incidence from 1999 to 2009 and PM2.5 found a statistically significant correlation with lung cancer and revealed that females had a slightly higher risk than did males [21]. However, few studies have analysed the effect of PM2.5 on lung cancer incidence in different regions of China. A work which explored the association between lung cancer mortality rate and PM2.5 in 31 provinces in China indicated that there were three northeastern provinces and some southern provinces that had positive correlations with lung cancer in males and females, respectively [48].

The chemical compositions of PM2.5 in China are mainly including organic matter, heavy metal, inorganic ion and trace inorganic non-metallic elements [49, 50]. Yao [51] indicated that the dominant ionic species SO2–4 in PM2.5 might be formed through the direct emissions of SO_2_. Our study found the interaction between SO_2_ and PM2.5 can modify the risk of lung cancer, no matter in males or females in north China. This is consistent with the spatial distribution of PM2.5 in China. Previous studies reported the average concentration of PM2.5 in north China reached as high as 80–100 μg/m^3^, while in south, the concentration has reduced to 40-70 μg/m^3^ [52].

The health effects of ambient O_3_ and CO have been assessed recently. One study used 20 communities (10 each from southern and northern U.S.) using a generalised linear model to estimate the association between increments in O_3_ concentration and mortality in every community, and found that the effect of O_3_ on mortality was positively related to latitude [53]. A study in Taiwan found a significant correlation between CO and O_3_, and female lung cancer [35]. In our study, CO and O_3_ had a significant effect on lung cancer, but they showed substantial discrepancy in north and south China. They were more strongly association with lung cancer in the south.

Some previous studies have indicated local regional relationships between PM10 or NO_2_ and lung cancer. One study in New Hampshire, U.S., found a significant contribution of PM10 to lung cancer only in the northern and southwestern regions of the state; thus, PM10 did not completely explain the lung cancer incidence [54]. Another study from Saudi Arabia analysed the association between NO_2_ and the incidence of the most common cancers (including lung cancer) and found higher coefficients of determination in the Eastern, Riyadh and Makkah regions than in other areas [55]. These findings are consistent with current study, which also indicated geospatial heterogeneity in the influence of PM10 and NO_2_ on lung cancer.

Meanwhile, our study found a stronger association between lung cancer and air pollution in males than in females in south China. This may be attributable to the fact that there is a high proportion of women suffering the risk of household pollution from such substances as pollutants emitted in the cooking of food [56]. Exposure to household pollution can pose a serious health hazard, particularly for women who spend much time inside of their house [57].

The potential biological mechanism behind the higher sensitivity of the south population than the north to outdoor air pollution might be that the north has a higher risk of household pollution [58], which is a top cause of death in China [14]. Household air pollution causes about 420,000 premature deaths annually, which is 40% more than that attributed to outdoor air pollution [59].

The combustion of solid fuels (biomass and coal) is the main source of household air pollution [59]. Approximately 72.5% of the population in the north uses solid fuels, which is 13% higher than that in the south. Compared with the winter temperature in the south, that in the north is lower, thus leading to prolonged heating times. This may be putting the north at risk of household air pollution. For example, a study which monitored three important indoor air pollutants (CO and respirable particles (RPM)) in four provinces during heating seasons in China reported that northern provinces have higher CO and RPM concentrations in rooms than do southern provinces [60].

The significant effects of smoking on lung cancer among men may be attributed to high prevalence of smoking in males. When smoking interacts with air pollutants—especially PM2.5 or O_3_—the risk of lung cancer increases significantly. This indicates that when air pollution and smoking work together, the likelihood of lung cancer shows non-linear growth, an issue that requires attention in both regions of China.

There were also some limitations in the study. First, the different time period of lung cancer and air pollution would introduce some uncertainties in the results. However, the long-term spatial relationship between air pollution and lung cancer is relatively stable and the influence on results would be rare in the Geodetector method due to the use of categorical data. As such, although the study did not analyse the effects of long-term exposure to air pollution on lung cancer, uncertainties will be diminished by using Geodetector method. Second, under-reporting or misdiagnosis of lung cancer datasets were not considered in this study. Although it may lead the misunderstanding results, this limitation can be attenuated by the examination of data for quality and integrity. Since the data was officially published by the National Cancer Center and reported under quality assurance procedures to guarantee its reliability, the possibility of under-reporting or misdiagnosis within the dataset is significantly reduced. Finally, since exposure to other pollutants such as airborne polycyclic aromatic hydrocarbons (PAHs) [61] or total suspended particle (TSP) [10] are also among the prominent causes of lung cancer, additional factors should be taken into account in future research.

## Conclusion

In conclusion, this study found apparent spatial and gender heterogeneities in the association between air pollution and lung cancer. Air pollution in south China has a greater impact on lung cancer than that in the north; however, females in the north are more likely to be affected by polluted air than the males. In north China, the main risk factors is SO_2_, and the dominant interactive factor is that between SO_2_ and PM2.5. Meanwhile, in south China, the six selected pollutants in this study have different degrees of influence on lung cancer, and the dominant interactive factor are that between SO_2_ and O_3_ in males and between SO_2_ and CO in females.

## Data Availability

The datasets used during the current study are publicly available. The lung cancer incidence data is available in the book: Jie He CW: *Annual Report of Cancer Registration in China 2016*. China: Tsinghua University Press; 2017*,* the air pollution data is available in http://106.37.208.233:20035/.

## Abbreviations

CO: Carbon monoxide
NO_2_: Nitrogen dioxide
O_3_: Ozone
PAHs: Airborne polycyclic aromatic hydrocarbons
PM10: Inhalable particles
PM2.5: Fine particulate
SO_2_: Sulfur dioxide
TSP: Total suspended particle
